# Electronic sow feeding: characterizing feeding patterns of gestating sows and their associations to reproductive performance

**DOI:** 10.1186/s13620-026-00345-3

**Published:** 2026-04-23

**Authors:** Mario Andre S. Ornelas, Edgar Garcia Manzanilla, Telmo Nunes, Maria Rodrigues da Costa

**Affiliations:** 1 Pig and Poultry Research and Knowledge Transfer Department, Teagasc Animal and Grassland Research and Innovation Centre, Moorepark, Fermoy, Cork, P61 C996 Ireland; 2 School of Veterinary Medicine, University College Dublin, Belfield, Dublin, Ireland; 3 CIISA – Centre for Interdisciplinary Research in Animal Health, Faculty of Veterinary Medicine, University of Lisbon, Lisbon, 1300-477 Portugal; 4 Centre for Epidemiology and Planetary Health, School of Veterinary Medicine and Bioscience, Scotland’s Rural College (SRUC), 9 Inverness Campus, Inverness, IV2 5NA Scotland, UK

**Keywords:** Animal welfare, Group-housing, Pig, Precision livestock farming, Swine

## Abstract

**Background:**

There is evidence that data recorded by electronic sow feeding (ESF) systems can generate useful information to support decision-making at farms. More specifically, healthy and diseased animals exhibit different patterns of feeding station usage. However, for feeding records to be used as a monitoring tool, it is necessary to understand feeding patterns, and how they differ across age groups and gestation stage, or how they change in response to disease. This study aimed to identify feeding patterns of gestating sows using ESF and to study their associations with parity and reproductive performance.

**Results:**

Feeding records of 276 group-housed sows in a dynamic group were analysed. These comprised 15 weeks of data for each sow. Visits made to ESF stations were mostly non-nutritive visits (60.01 ± 19.8%), and daily rations were consumed predominantly in a single visit (98.3 ± 1.7% of all individual feeding cycles). Animals displayed a clear preference for a specific feeding station and the number of non-nutritive visits made was negatively related to gestation week (b = -0.067) and parity (b = -0.230) (*P* < 0.001). Despite the dynamic nature of the group, sows kept the feeding order relatively stable over time, especially among those feeding first. Moreover, the odds of a sow being among the first 15% to eat increased with every additional parity [OR: 2.16, *P* < 0.010]. Concerning reproductive performance, pre-weaning piglet mortality was negatively associated with average feeding time (*P* = 0.011).

**Conclusions:**

The analysis of ESF data revealed evident patterns in the use of feeding stations by sows. These patterns varied according to parity and gestation stage and were associated with reproductive performance. Further research characterizing the feeding behaviour of gestating sows is necessary to support the implementation of ESF records as a monitoring tool for group-housed sows.

## Background

Since its creation in the 1980’s, electronic sow feeding (ESF) has become a widely adopted system to manage group-housed sows, as it allows each animal to be fed according to its requirements, during gestation [1–3]. The introduction of EU legislation banning sow gestation stalls in 2013 [4] led to a further increase in the adoption of ESF systems. California’s Proposition 12 [5] is also encouraging the elimination of gestation stalls in several U.S. states and may therefore further promote the adoption of ESF systems. Besides enabling control over feed intake, thus optimizing conditioning for reproductive performance, these systems generate a series of records every time a sow visits a feeding station. Not only is this a completely automated and continuous process, but data are collected at the individual level, making it possible to characterize the feeding behaviour of each sow [6–8]. Despite this, the value of feeding records as a monitoring tool is still overlooked and, as a result, they are seldom used for decision-making at farms. An exception to this is the automatic identification of animals with a decreased feed intake [6, 7].

After processed, data collected at feeding stations can be used to study how often a sow visits a station, daily feed intake, preferred feeding time or the order in which animals feed. Previous works succeeded in detecting lame sows [9] or returns to oestrus [7] by monitoring their feeding order. Indeed, the existence of stable feeding and milking orders have been described in pigs [10] and dairy cows [11], respectively, and deviations from these have been associated with disease. Other examples include associations between time spent in ESF stations and gestation losses [12] and between number of non-nutritive visits to ESF stations and animal welfare indicators [13].

To effectively use ESF records for early detection of sows needing special attention, it is necessary to understand how the use of feeding stations varies according to factors like parity, gestation stage and health. Electronic sow feeding systems are a powerful tool to detect these patterns and to notify farmers of any deviations. The aims of the present study were (1) to characterize patterns in the use of ESF stations by sows according to parity and gestation stage and (2) to study associations between different patterns and reproductive performance.

## Methods

### Animal housing and management

The study took place in a 200-sow farrow-to-finish high health status farm, with a 3-week farrowing batch system. The gestating sows were kept as one dynamic group of approximately 120 individuals, with access to two fully protected forward-exit ESF stations (Schauer Compident VI, Schauer Agrotonic GmbH, Prambachkirchen, Austria). The group was dynamic rather than static, meaning that its composition changed periodically, with recently inseminated animals and animals due to farrow soon being introduced and removed from the group, respectively. A feeding cycle comprehended a 23-hour interval starting at 17:00, during which sows could enter a vacant station to consume their allotment, in one or multiple visits. Feeding level was restricted and each animal was assigned to one of the following feeding curves: (a) 2.2 kg/day until day 90 of gestation and 2.6 kg/day thereafter or (b) 2 kg/day throughout the whole gestation.

### Data processing

A compilation of ESF records concerning a one-year period (364 feeding cycles) was studied. Each record corresponded to a feeding station visit and it included station ID, sow ID, feed intake value (amount eaten at each visit, presented in kg), date, time of entry and time of exit. According to the feed intake values, ESF visits were classified as “non-nutritive” (NNV; intake = 0 kg) or “nutritive” and feed intake was calculated as the total amount eaten per sow for every feeding cycle.

The study unit consisted of one sow-gestation, as opposed to one sow. For animals with two gestations over the study period, each gestation counted as one study unit. Gilts were excluded once they were housed on a different pen and were subjected to a training process on ESF utilization before being introduced to the dynamic group. Sow parity and individual reproductive performance were retrieved from the farm’s management software and collated to the ESF records. Reproductive performance data of each sow, including the number of piglets born alive (TBA), average litter birth weight (ABW), and pre-weaning mortality (PWM) were characterized and studied. Out of the 276 sow-gestations included in the analysis, 268 had data for TBA and ABW while PWM was available for 243 sow-gestations.

### Descriptive analysis and statistical analysis

All data was processed using Microsoft Excel^®^ and further analyses were performed using R version 4.5.2 [14], including R packages ggplot2 version 3.5.2 [15] and lme4 version 1.1.37 [16]. Statistical significance was determined using an alpha level of 0.05 and results are expressed as mean ± standard deviation.

The number and type of feeding station visits across gestation were computed and analysed for every sow-gestation, along with feeding times and feeding rates. Because feeding level was restricted and different between individuals, comparisons based solely on feed intake or meal duration were not conducted. Further, ESF systems are unable to determine the time a sow initiates or concludes a meal. Thus, individual feeding rates were estimated as the ratio between total feed dispensed to a sow during gestation and total time spent performing nutritive visits. To study how the number of NNV varied across gestation, a generalized linear mixed effects model was fitted, based on a Poisson distribution. Gestation week and parity were included as fixed effects, while random effects included intercepts for subjects and by-subject random slopes for the effect of gestation week. To assess individual feeding station preference, the most visited station of each animal was determined, and the percentage of nutritive visits made in the preferred station was calculated based on the total number of days with access to the ESF stations. For every sow-gestation, feeding times were classified based on their temporal consistency across gestation, which relied on the percentage of meals eaten within a timeframe of 5 h (average feeding time 2.5 h). Percentages greater than or equal to 90%, not exceeding 70% and between these two ranges were considered regular, moderately regular and irregular, respectively. For sows performing several nutritive visits on a feeding cycle, the time of the first visit was considered.

To assess the stability of the feeding order, the position of every sow in the feeding order was computed across gestation and the 18 individuals that more frequently occupied the first 15% and last 15% positions were selected. In order to study the associations between feeding order and use of feeding stations, a logistic regression model was carried out, with feeding rank, either first 15% or last 15% to feed, as the dependent variable. It included parity, feeding rate and total number of feeding station visits as continuous explanatory variables.

Beta regression models were used to assess if feeding station preference was associated with parity, average feeding time and total number of NNV, using the normalized percentage of days on the preferred feeder as the response variable. Associations between feeding patterns and reproductive performance were assessed through linear regression modelling. Unlike TBA and ABW, PWM did not follow a normal distribution and thus was modelled using beta regression (*betareg* function from betareg package in R [17]). For each response variable, univariable and multivariable analyses were conducted using parity class and average feeding time as explanatory variables. Parity class consisted of a categorical variable with two levels: parity ≤ 2 and parity ≥ 3.

## Results

### Use of ESF stations

A total of 391 sow-gestations were identified, of which 276 were considered complete (i.e. with at least 100 feeding cycles) and included in the study. Of these, 95 sows had two gestations and 86 sows had one gestation. The parity of the study group was 4 ± 2.3.

Over the length of 364 feeding cycles, 101,493 ESF station visits were recorded from the 276 studied sow-gestations, corresponding to 28,931 individual cycles (Table 1). While there was a small variation on total nutritive visits between animals (103.9 ± 2.2), total NNV differed substantially (263.8 ± 282.6). Most sows consumed their daily allotment in a single visit (98.3 ± 1.7% of individual feeding cycles) and did so at a feeding rate of 189 ± 34 g/min. With regards to number of daily visits per individual, animals performed 2.52 ± 3.85 NNV and 0.99 ± 0.14 nutritive visits per day.

**Table 1:** Individual use of electronic sow feeding stations

Parameter	Values per sow-gestation Mean (± SD)
Number of feeding cycles - (data available per sow-gestation)	104.8 (± 0.9)
Total ESF visits - of which nutritive visits (%)	367.7 (± 282.8) 39.9 (± 19.8)
Percentage of daily meals eaten in a single visit (%)	98.3 (± 1.7)
Percentage of meals eaten in the preferred feeding station (%)	89.2 ± 12.6%
Feeding rate (g/min)	189 ± 34

### Number and time of non-nutritive visits

**Fig. 1:**
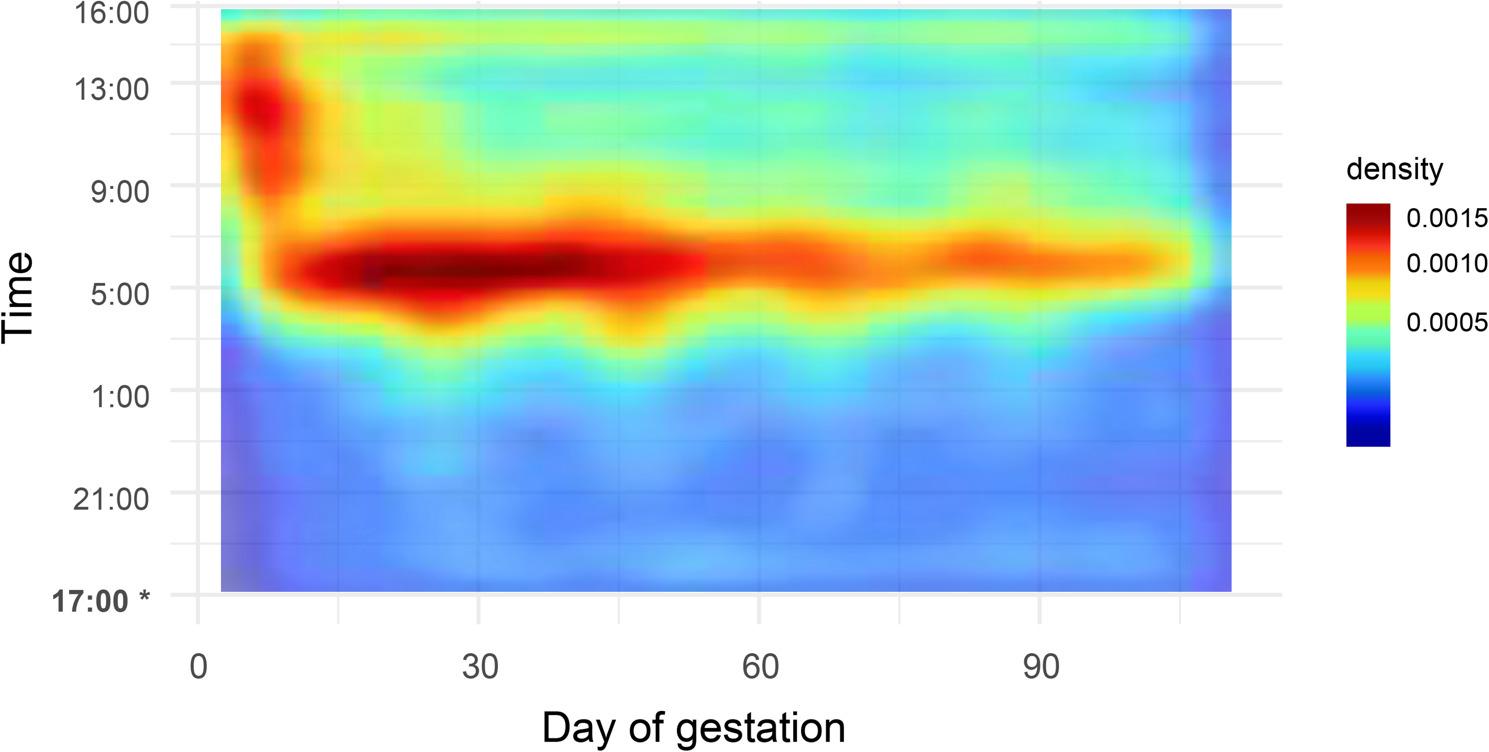
Circadian distribution of non-nutritive visits across gestation for the studied sow-gestations based on Kernel density estimation. *Each feeding cycle started at 17:00 and finished at 16:00

When considering all sows, it was observed that as gestation advanced the number of NNV decreased and there was a time span of nearly 4 h (04:00 - 08:00) when most visits took place (Fig. 1). In the fitted mixed effects model, both gestation week (b = -0.067, SE = 0.004, *P* < 0.001) and parity (b = -0.230, SE = 0.022, *P* < 0.001) were significant explanatory variables of the number of NNV.

### Feeding times

**Fig. 2:**
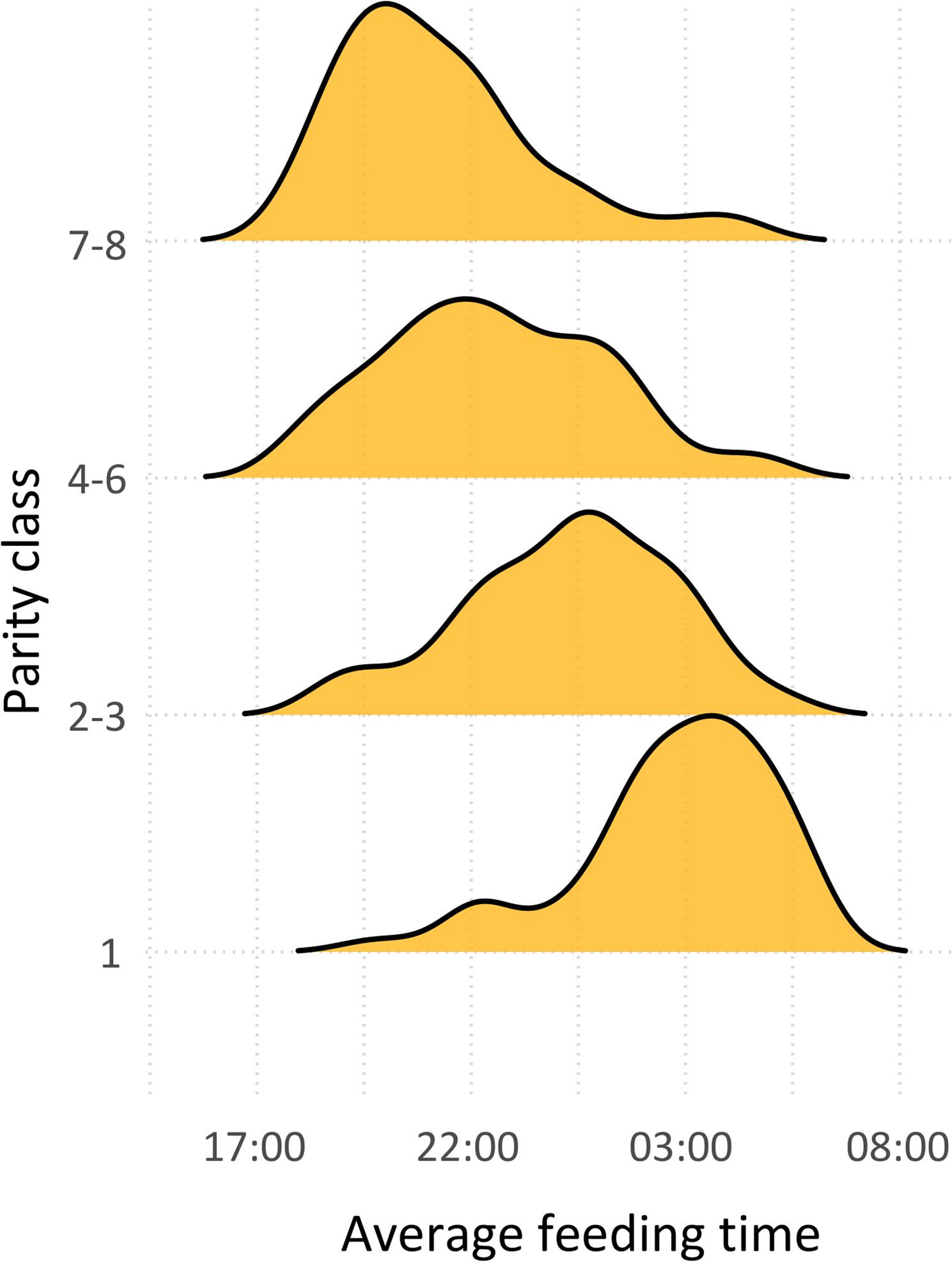
Average feeding time across gestation according to parity class. Note: a feeding cycle comprehended a 23-hour interval starting at 17:00

**Fig. 3:**
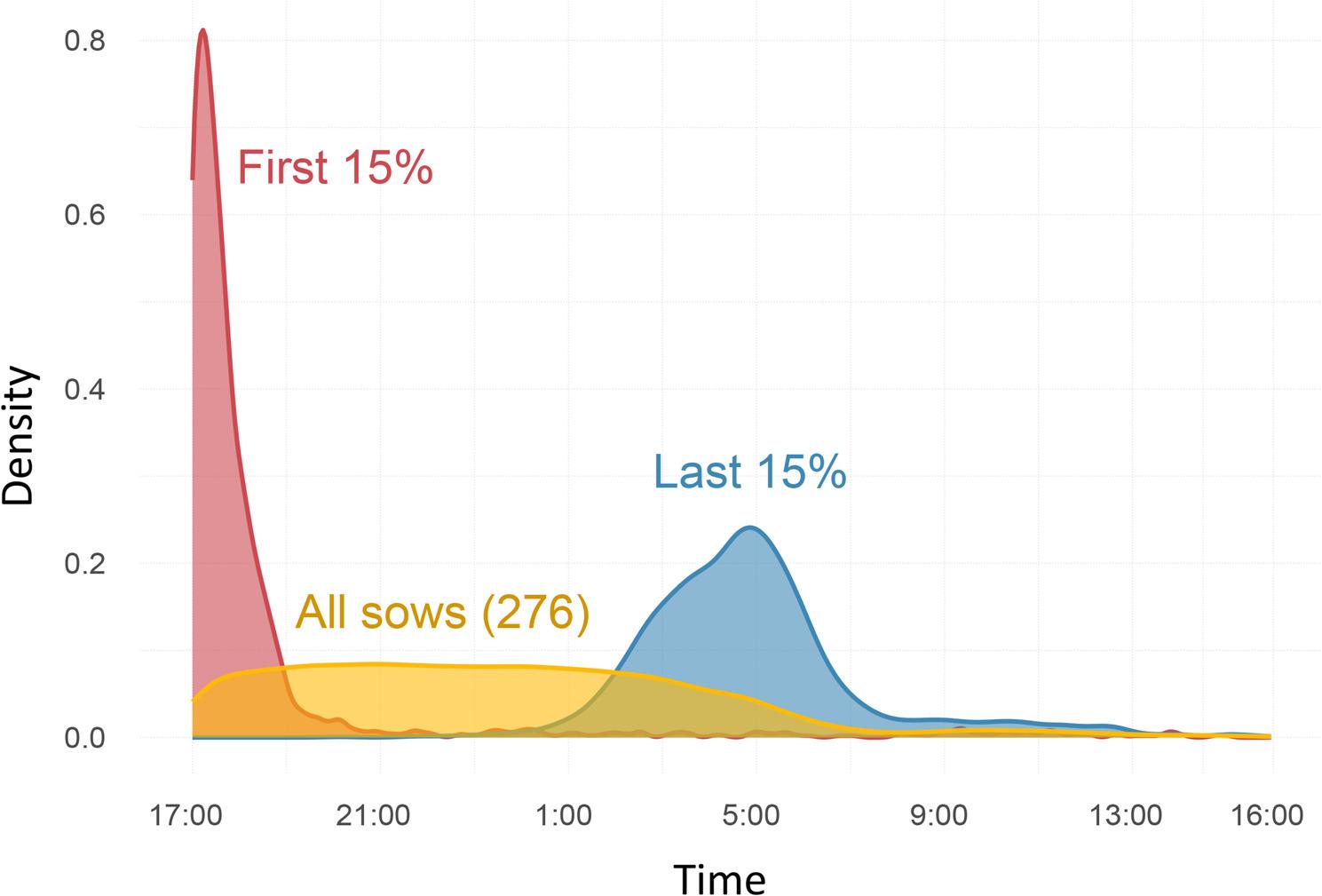
Circadian distribution of feeding times according to feeding order of the first 15% sows to feed, last 15% sows to feed and the entire study group (*N*=276) based on Kernel density estimation

Average feeding time was positively associated with parity, as shown in Fig. 2. Feeding activity was highly concentrated during the first half of feeding cycles (17:00 - 05:00). The first 15% of sows to feed, consumed their meal within the first 2 h of feeding cycles whereas the last 15% to feed displayed greater variability (Fig. 3). This last group fed mainly during night time, approximately 9 to 14 h after the feeding cycle start time. With regards to temporal consistency of feeding times, 67 sows were classified as displaying regular feeding times (24.3%), 64 as irregular (23.2%) and 145 as moderately regular (52.5%).

### Feeding station preference

**Fig. 4:**
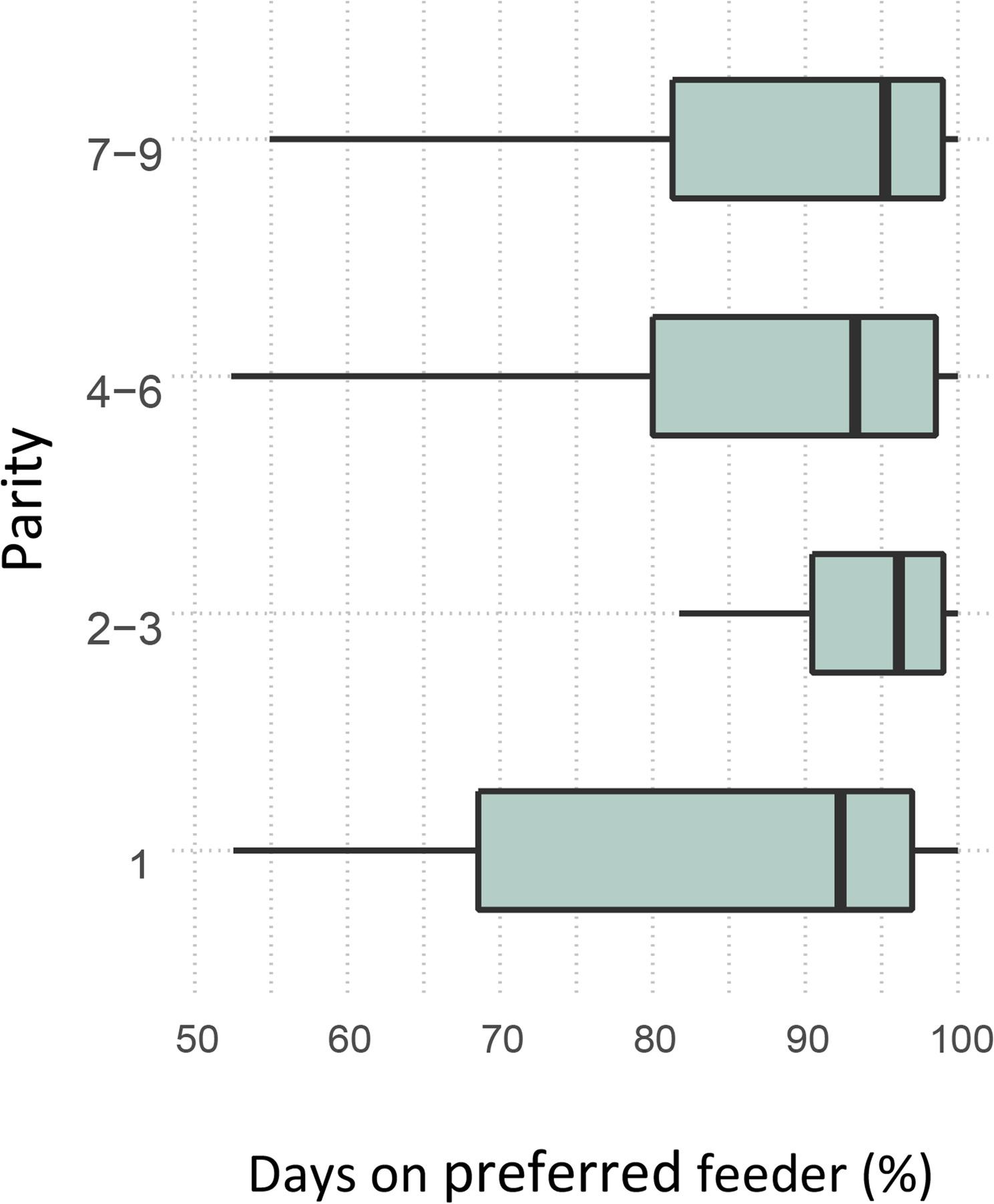
Feeding station preference by parity, calculated as the percentage of days on which each sow obtained the daily ration from its preferred station. Animals were grouped into four parity classes: 1 (*n* = 49), 2-3 (*n* = 76), 4-6 (*n* = 63) and 7-9 (*n* = 88)

There was an evident preference for a particular feeding station. On average, sows chose their preferred feeding station on 89.2 ± 12.6% of occasions (Fig. 4). Further, 13% of the study group (*n* = 36) fed from the preferred feeding station on every nutritive visit across gestation. Feeding station preference was associated with average feeding time (β = -0.067, SE = 0.020, *P* < 0.001) and total number of NNV (β = 0.001, SE = 0.0002, *P* = 0.005), but not with parity. The total number of nutritive visits was evenly distributed by the two feeding stations (54% and 46%). Likewise, for non-nutritive visits, the use rate of each feeding station was 57% and 43%.

### Feeding order

Due to the dynamic nature of the group, the feeding order of sows was not stagnant. Despite this, the first 15% sows to feed occupied that position on 89.7 ± 4.27% of the days and the last 15% to feed did so on 69.8 ± 10.2% of occasions. It was estimated that the probability of a sow being among the first 15% to feed was positively related to parity [OR: 2.16, *P* < 0.010], but not associated with the total number of ESF visits or feeding rate.

### Sow reproductive performance

According to univariable analyses, both parity (*P* = 0.002) and average feeding time (*P* = 0.011) were associated with PWM, as older sows and sows that fed earlier had higher mortality rates. When both variables were included in multivariable analysis of PWM, only parity was statistically significant (*P* = 0.022). In contrast, TBA and ABW were not associated with parity or feeding time.

## Discussion

Even though ESF systems have existed for nearly four decades, our understanding of the feeding behaviour of gestating sows in modern ESF systems is still limited. This study addresses this gap and provides useful information on the management of ESF systems to reduce feeding competition.

### Number and type of ESF visits

Throughout the 15 weeks that each sow spent group-housed, the total number of ESF visits differed considerably between individuals (367.7 ± 282.8), most of which were NNV (60.01 ± 19.8%). In contrast, nutritive visits were more consistent in number with nearly all sows consuming their daily allotment in a single visit. Previous studies using ESF systems [7, 18] have reported high proportions of sows eating their ration in a single visit (72.25% − 83.88% and 79.5% − 95.3%, respectively). Cornou et al. [7] and Søllested [18] further described that 60.8% − 68.8% of all visits were NNV, which is in line with the figures of the current study. In addition, the mixed effects model fitted to investigate how the number of NNV was related to time and parity, suggested that sows tended to visit feeding stations less frequently as gestation advances, and younger sows (low parity) are more likely to make more visits than older ones (high parity). A recent study by Lagoda et al. also reported a decline in the number of NNV throughout gestation in a group of gilts [13]. In contrast, Søllested [18] and Chapinal [19] found no association between time and number of visits, although the latter observed a decrease in feeder occupancy using an unprotected feeding station. The results of the present study suggest that experienced sows (at the end of gestation or higher parities) have learnt that it is not worth visiting the feeding station again if they already had their daily meal, as feeding level is restricted. The clear individual preference of sows for a specific feeding station may reflect a social mechanism to avoid competition, as suggested by Eddison [20], who also reported a marked preference for feeding stations.

### Feeding order

Given the dynamic nature of the study group, the presence of a highly stable feeding order was not expected. Despite this, sows accessed feeding stations in a relatively stable order during each feeding cycle, especially among those feeding first. The fact that 24.3% and 52.5% of animals consumed their daily allotment within a 5-hour timeframe on at least 90% and 70–90% of occasions, respectively, is also an indicator of stability in the feeding order. These results are consistent with those from previous works reporting different indicators of stability in the feeding order, in static [19, 21–23] and dynamic [24] groups. In fact, as early as 1988, authors analysed the position of each sow in the feeding order over a week, describing that individuals in the bottom half of the order had higher standard deviations compared with those feeding earlier [25]. In the present study it was also observed that with each additional parity, the odds of a sow belonging to the first 15% group members to feed increased 2.16 times, resembling the findings of Hunter et al. [25] and Strawford et al. [26]. This was expected as social rank, which is associated with parity, is often estimated using feeding order as a proxy [13, 22, 23, 27–29].

### Reproductive performance

In order to investigate associations between feeding patterns and reproductive performance, average feeding time was used as a proxy for feeding competition and included in the analyses as an explanatory variable. According to univariable analysis, average feeding time was negatively associated with PWM, while parity was positively associated with PWM. However, in multivariable analysis only parity was a significant explanatory variable. Fig. 2 shows a strong negative association between parity and average feeding time, thus suggesting a confounding effect in multivariable analysis. Due to the limited sample size in the present study, the assessment of the contribution of average feeding time to PWM was compromised, as well as the potential interaction between average feeding time and parity.

Being a sequential system where animals largely outnumber feeding places, ESF gives rise to competition over access to feeding stations. This is particularly evident following the start of feeding cycles and often results in increased aggressive interactions [10, 30, 31]. In the current study, the first 15% and last 15% of sows to feed were compared to assess the stability of the feeding order. As shown in Fig. 3, the first 15% animals to feed did so within the first 2 h of each cycle, thus being repeatedly exposed to a stressful competitive environment. Presumably, the same did not happen for sows feeding last during night time after the rest of the group had fed. While the potential association between feeding time, as a proxy of feeding competition, and PWM may reflect the different social stress conditions that sows were exposed to during gestation, it should be mentioned that associations between feeding behaviour and reproductive performance are not well understood. In a study by Iida et al., sows that spent less time in feeding stations had higher gestation losses [6]. Kranendonk et al. reported that sows more frequently displaced at the feeder (with a low social rank) had litter characteristics similar to those of high ranked group members, except for having lower weaning weights [32].

In the present study, feeding rate was not associated with parity or average feeding time, and did not differ between the first 15% and last 15% of sows to feed. Interestingly, Vargovic et al. observed that sows with a higher feed consumption rate or with irregular feeding patterns performed worse, having more farrowing failures and early sow culling [33]. It is worth mentioning that ESF records do not allow an accurate estimation of feeding rate. While the time that a sow enters a feeding station is a reliable indicator of when it starts feeding, the time it exits a station does not necessarily indicate when it stops feeding. In this study, all animals were given 15 min per nutritive visit to consume their daily allotment before feed bowl was retracted and another animal was let inside the feeding station. Therefore, it cannot be assumed that the duration of a nutritive visit corresponded to the duration of a meal. Further, imposing a time limit for feeding can affect feeding behaviour, as sows have been observed to eat more slowly in the absence of a time restriction [33].

While there have been several studies comparing reproductive performance between sows fed with ESF and other common feeding systems for group-housed sows, current knowledge on how ESF data can be used to detect health and welfare issues is limited [34–39]. Based on our results, some recommendations can be made to stakeholders using ESF systems. Because competition to access feeding stations may impact sow health and welfare, including reproductive performance, adopting certain measures to mitigate such competition, like increasing the number of stations, might be beneficial. A critical yet commonly overlooked measure is the strategic display of feeding stations, ensuring that sows leaving a station are unable to promptly access the same or another station’s entrance. Indeed, the design of the pen plays an important role on the feeding behaviour of animals as well as their performance. Other methods may include setting the feeding cycle to start later in the evening, as proposed by Jensen et al. [31], who observed that overnight feeding resulted in improved behaviour.

Finally, it became evident with this work that even though ESF software gathers large amounts of data, it presents information in a rather limited way. With the technological revolution of livestock farming, the use of data to generate meaningful information with actionable insights for farmers is crucial. This study contributes to a better understanding of how electronic feeding records can be used to improve sow welfare and, consequently, reproductive performance. However, an important gap remains in how data is presented, as information generated by ESF records is not easily accessible and relatable for farmers to support their day-to-day decision making.

## Conclusion

The analysis of ESF data revealed evident patterns in the use of feeding stations by sows. Animals displayed a clear preference for a specific feeding station, making fewer non-nutritive visits as gestation progressed and as parity increased. A relatively stable feeding order was also observed, especially among sows feeding first, which were also generally older and had higher PWM. The results reflect the competitive feeding environment consistently described in previous ESF studies, underscoring the importance of adequate design and integration of ESF systems in sow pens. This study emphasizes the value of ESF records as individual monitoring tools, thus highlighting their potential as a component of precision livestock farming. Nevertheless, further characterizations of feeding patterns and improvements on the display of information are necessary to support the implementation of ESF records as a monitoring tool for group-housed sows.

## Data Availability

The datasets used and/or analysed during the current study are available from the corresponding author on reasonable request.

## Abbreviations

ABW: Average birth weight
ESF: Electronic sow feeding
NNV: Non-nutritive visits
OR: Odds ratio
PWM: Pre-weaning mortality
TBA: Number of piglets born alive
